# Dual contrast enhanced cardiac MRI using manganese and gadolinium in patients with severe ischemic cardiomyopathy detects the peri-infarct region (PIR)

**DOI:** 10.1186/1532-429X-16-S1-O96

**Published:** 2014-01-16

**Authors:** Yuka Matsuura, Rajesh Dash, Paul J Kim, Hadas Shiran, Aparna Bhagavat, Phillip Harnish, Michael V McConnell, Phillip Yang

**Affiliations:** 1 Cardiovascular Medicine, Stanford University Medical Center, Stanford, California, USA; 2 San Mateo Medical Center, San Mateo, California, USA; 3 Eagle Vision Pharmaceutical Corporation, Exton, Pennsylvania, USA

## Background

Delayed Enhanced MRI (DEMRI) with gadolinium (Gd) is used as gold standard for diagnosis of myocardial infarction. However, the non-specific property of Gd overestimates the infarct size. Conversely, manganese (Mn2+) enters only the live, active cardiomyocytes via L-type Ca2+ channels. From our earlier work in animal MI models, manganese-enhanced MRI (MEMRI) has demonstrated its utility in identifying the viable, non-viable, and injured myocardium. We performed the "first in human" dual-contrast MEMRI-DEMRI to assess the efficacy of MEMRI-DEMRI to identify the peri-infarct region (PIR) in patients with severe ischemic cardiomyopathy (ICM).

## Methods

5 ICM patients (Class I-III CHF) have been enrolled (5 male, mean age 60 ± 7 years). Cardiac MRI was performed using a 3.0T MRI scanner (Signa 3T HDx, GE HealthCare, USA) with an 8 channel cardiac coil (3.0T HD Cardiac Array, GE HealthCare, USA). LV functional images and DEMRI were acquired on the first day of this study, and MEMRI was acquired on the following week. (1) LV function: SSFP, flip angle (FA) 45, slice thickness (ST) 8.0 mm, matrix 224 × 224, FOV 35.0 cm; (2) DEMRI: FGRE-IR, TR 6.0, TE 2.8, TI 200-300, FA 15, ST 8.0 mm, matrix 224 × 192, FOV 35.0 cm, 0.2 mmol/kg Gd (Magnevist, Bayer HealthCare, Germany); and (3) MEMRI: FGRE-IR, TR 6.0, TE 2.8, TI 600-700, FA 15, ST 8.0 mm, matrix 224 × 192, FOV 35.0 cm, 1 mmol/kg SeeMore (Eagle Vision Pharmaceutical, USA) were performed. The infarct volumes were determined as 3 standard deviations (SDs) above mean on DEMRI and 2 SDs below mean on MEMRI using a Cardiac MRI Software (CMR42, Circle Cardiovascular Imaging Inc., Canada).

## Results

**Figure 1:**
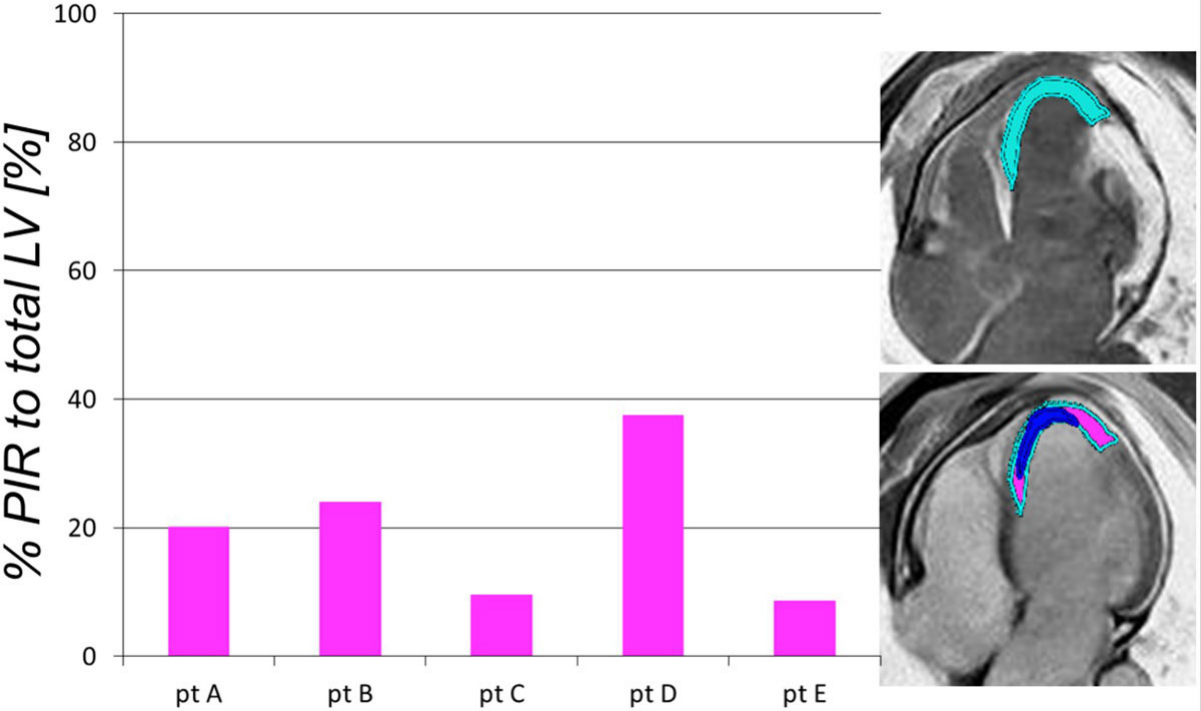
**The percentage Peri-Infarct Region (PIR) per total LV volume**.

**Figure 2:**
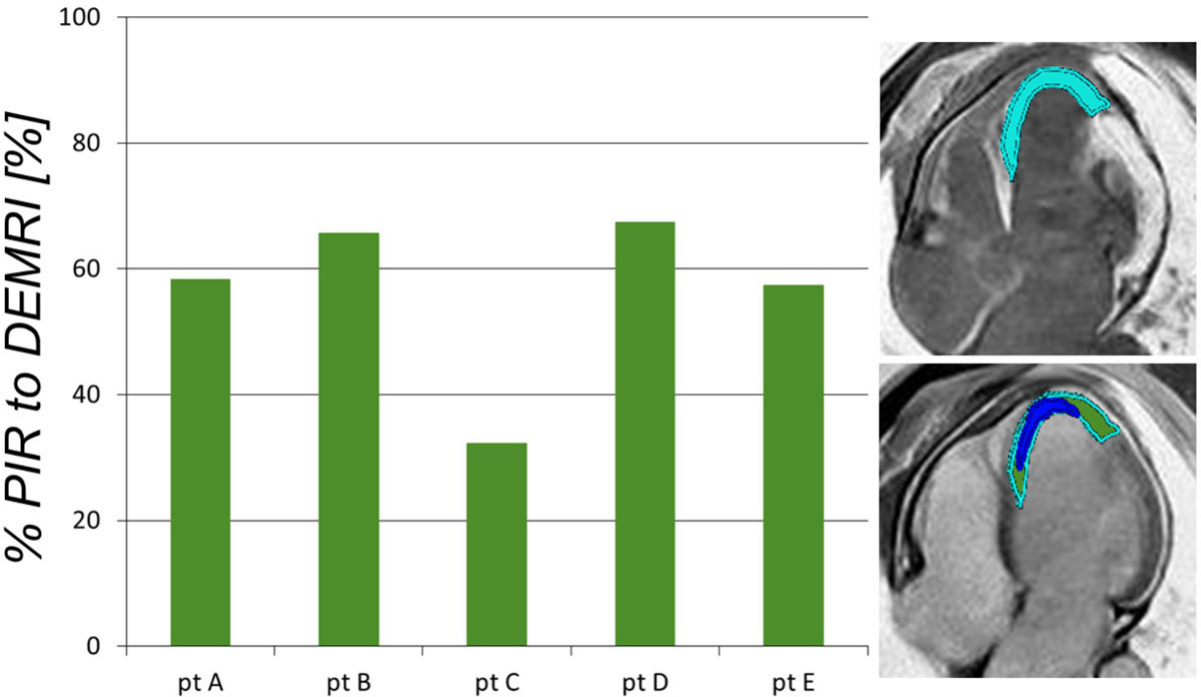
**The percentage Peri-Infarct Region (PIR) per DEMRI enhanced volume**.

The average LVEF was 35 ± 4%. The % enhanced DEMRI infarct volume (34 ± 11%*) was significantly (*p < 0.05) higher than the % defect MEMRI infarct volume (14 ± 3%). The PIR was calculated as the difference between DEMRI and MERMRI. The mean % PIR per total LV and the mean % PIR per DEMRI enhancement were 20 ± 12% (Figure 1) and 56 ± 16% (Figure 2), respectively.

## Conclusions

The non-viable myocardium volume, appearing as MEMRI defect, was significantly smaller than the DEMRI enhancement. The discrepancy between DEMRI and MEMRI may represent the PIR or "area-at-risk". Therefore, our results suggest that the dual MEMRI-DEMRI contrast may clearly delineate the PIR by integrating the biology of viable myocardium and anatomy of non-viable myocardium. Further studies on the ability of this dual contrast approach to delineate the area-at-risk and to predict clinical outcomes from revascularization are necessary.

## Funding

Stanford University fellowship of Astellas Foundation for Research on Metabolic Disorders (YM).

